# Supplementary material for: Smartphone-supported Positive Adjustment Coping Intervention (PACI) for couples undergoing fertility treatment: A randomised controlled trial
Source: PLoS One. 2025 Dec 8;20(12):e0335776. doi: 10.1371/journal.pone.0335776 (PMC12685219; doi:10.1371/journal.pone.0335776)
Supplement: S1 File — Reporting checklist for randomised trial. Based on the CONSORT guidelines. (PDF) [file pone.0335776.s001.pdf]

Reporting checklist for randomised trial.

S1 File. Consort checklist. Reporting checklist for randomised trial. Based on the CONSORT guidelines.

		Reporting Item	Page Number
**Title and Abstract**			
Title	#1a	Identification as a randomized trial in the title.	1
Abstract	#1b	Structured summary of trial design, methods, results, and conclusions	2
**Introduction**			
Background and objectives	#2a	Scientific background and explanation of rationale	3-4
Background and objectives	#2b	Specific objectives or hypothesis	4-5
**Methods**			
Trial design	#3a	Description of trial design (such as parallel, factorial) including allocation ratio.	5
Trial design	#3b	Important changes to methods after trial commencement (such as eligibility criteria), with reasons	Not applicable
Participants	#4a	Eligibility criteria for participants	6-7
Participants	#4b	Settings and locations where the data were collected	6
Interventions	#5	The experimental and control interventions for each group with sufficient details to allow replication, including how and when they were actually administered	8
Outcomes	#6a	Completely defined prespecified primary and secondary outcome measures, including how and when they were assessed	8-9

Outcomes	#6b	Any changes to trial outcomes after the trial commenced, with reasons	Not applicable
Sample size	#7a	How sample size was determined.	7
Sample size	#7b	When applicable, explanation of any interim analyses and stopping guidelines	Not applicable
Randomization - Sequence generation	#8a	Method used to generate the random allocation sequence.	7
Randomization - Sequence generation	#8b	Type of randomization; details of any restriction (such as blocking and block size)	7
Randomization - Allocation concealment mechanism	#9	Mechanism used to implement the random allocation sequence (such as sequentially numbered containers), describing any steps taken to conceal the sequence until interventions were assigned	7
Randomization - Implementation	#10	Who generated the allocation sequence, who enrolled participants, and who assigned participants to interventions	7
Blinding	#11a	If done, who was blinded after assignment to interventions (for example, participants, care providers, those assessing outcomes) and how.	7
Blinding	#11b	If relevant, description of the similarity of interventions	4
Statistical methods	#12a	Statistical methods used to compare groups for primary and secondary outcomes	10-11
Statistical methods	#12b	Methods for additional analyses, such as subgroup analyses and adjusted analyses	10-11
**Results**			
Participant flow diagram (strongly recommended)	#13a	For each group, the numbers of participants who were randomly assigned, received intended treatment, and were analysed for the primary outcome	6-7, Table 1
Participant flow	#13b	For each group, losses and exclusions after randomization, together with reason	6-7, Figure 1

Recruitment	#14a	Dates defining the periods of recruitment and follow-up	6-7<br>Figure 1
Recruitment	#14b	Why the trial ended or was stopped	Protocol
Baseline data	#15	A table showing baseline demographic and clinical characteristics for each group	Table 1 & 2
Numbers analysed	#16	For each group, number of participants (denominator) included in each analysis and whether the analysis was by original assigned groups	Figure 1
Outcomes and estimation	#17a	For each primary and secondary outcome, results for each group, and the estimated effect size and its precision (such as 95% confidence interval)	13-15
Outcomes and estimation	#17b	For binary outcomes, presentation of both absolute and relative effect sizes is recommended	16,<br>Tables 5 & 6
Ancillary analyses	#18	Results of any other analyses performed, including subgroup analyses and adjusted analyses, distinguishing pre-specified from exploratory	16-18
Harms	#19	All important harms or unintended effects in each group	11
**Discussion**			
Limitations	#20	Trial limitations, addressing sources of potential bias, imprecision, and, if relevant, multiplicity of analyses	21-22
Generalisability	#21	Generalisability (external validity, applicability) of the trial findings	19-20
Interpretation	#22	Interpretation consistent with results, balancing benefits and harms, and considering other relevant evidence	19-20
Registration	#23	Registration number and name of trial registry	3
**Other information**			
Interpretation	#22	Interpretation consistent with results, balancing benefits and harms, and considering other relevant evidence	19-20

Registration	#23	Registration number and name of trial registry	3
Protocol	#24	Where the full trial protocol can be accessed, if available	~~S2 File~~
Funding	#25	Sources of funding and other support (such as supply of drugs), role of funders	22
