# Supplementary material for: Smartphone-supported Positive Adjustment Coping Intervention (PACI) for couples undergoing fertility treatment: A randomised controlled trial
Source: PLoS One. 2025 Dec 8;20(12):e0335776. doi: 10.1371/journal.pone.0335776 (PMC12685219; doi:10.1371/journal.pone.0335776)
Supplement: Attachment — Submitted filename: Response to Reviewers.docx [file pone.0335776.s004.docx]

**Response to Reviewers**

*We would like to thank both Reviewers once again. We believe that the revisions prompted by the comments have substantially improved the manuscript.*

Review Comments to the Author

Reviewer #1: Table 2: Based on CONSORT statement/requirement, the column of t/X^2 and p value and the *P ≤ .05 in the footnote are to be removed.

For paired samples, Wilcoxon signed-rank test is to be mentioned.

*Response*: *Thanks for pointing that out. We removed the column of t/X^2 and p value and the *P ≤ .05 in the footnote. We have added the missing information (page 14, line 260).*
